# Post‐dural puncture headache: a prospective study on incidence, risk factors, and clinical characterization of 285 consecutive procedures

**DOI:** 10.1186/s12883-022-02785-0

**Published:** 2022-07-14

**Authors:** Jasem Al-Hashel, Azza Rady, Fathi Massoud, Ismail Ibrahim Ismail

**Affiliations:** 1 Department of Neurology, Ibn Sina Hospital, Kuwait City, Kuwait; 2 Department of Medicine, Faculty of Medicine, Health Sciences Centre, Kuwait University, Kuwait City, Kuwait

**Keywords:** Headache, Lumbar puncture, Post-dural puncture headache, Incidence, Risk factors

## Abstract

**Background:**

Lumbar puncture (LP) is a common and relatively safe neurological procedure. It can be complicated by post-dural puncture headache (PDPH) after both diagnostic and therapeutic procedures. The aim of this study is to identify the incidence, risk factors and clinical characterization of PDPH in the inpatient setting of the main tertiary neurology hospital in Kuwait.

**Methods:**

We conducted a prospective observational cohort study that included patients who were admitted to neurology department at Ibn Sina hospital, Kuwait, from January 1, 2019 to December 31, 2020, on whom, LP was performed for diagnostic and/or therapeutic reasons. Multivariate logistic regression analysis was performed to evaluate the association between PDPH and different clinical parameters.

**Results:**

A total of 285 patients were included; 225 females (78.9%), mean age of 32.9 ± 11.7 years. PDPH was reported by 84 patients (29.5%), with mean headache onset of 1.7 ± 0.8 days, and mean duration of 2.4 ± 2.1 days. The commonest headache type was dull aching in 49 patients (58.3%). Headache severity was mild to moderate in 64 patients (76.2%), with mean NRS of 4.1 ± 0.9. Most PDPH (99.3%) resolved with conservative medical management, with only 2 patients (0.7%) requiring epidural blood patch. In multivariate logistic regression model, there was a statistically significant correlation between development of PDPH and young age (*p* = 0.001), female gender (*p* = 0 .001), low BMI (*p* < 0 .001), pre-LP headache (*p* = 0.001), history of previous PDPH (*p* = 0.001), and number of LP attempts (*p* < 0.001). PDPH was statistically significantly higher in patients with optic neuritis (*p* = 0.009), and cerebral venous thrombosis (*p* = 0.007), and lower in patients with peripheral neuropathy (*p* = 0.011) and spinal muscular atrophy (*p* = 0.042).

**Conclusions:**

Findings from clinical practice in the main tertiary neurology hospital in Kuwait were in line with literature findings. Younger age, female gender, lower BMI, pre-procedural headache, previous history of PDPH, and number of LP attempts were found to be independent risk factors for developing PDPH. To our knowledge, this study represents the first comprehensive description of PDPH in a population from the Arabian Gulf Region.

## Background

Post-dural puncture headache (PDPH), formerly known as post-lumbar puncture headache, is a well-known adverse event that follows diagnostic and/or therapeutic puncture of the dura, or accidentally, following spinal anesthesia [1]. The classical features of PDPH according to the International Classification of Headache Disorders, 3^rd^ edition (ICHD-3) include headache that occurs within 5 days of lumbar puncture (LP), that is aggravated with standing or sitting position and relieved with lying down, and remits spontaneously within 2 weeks, or after sealing of the leak with epidural lumbar patch [2].

This particular type of headache has been an area of interest to physicians from different specialties, and several clinical studies have attempted to identify procedure- and non-procedure-related risk factors in the literature. Several modifiable and non-modifiable independent risk factors for PDPH have been documented in both anesthesiology and neurology studies. Class I and II evidence regarding the development of PDPH in literature included; female gender, age between 20 and 50 years, lower body mass index (BMI), previous history of PDPH, larger needle diameter, use of cutting needles, perpendicular insertion of the needle bevel to the long axis of the spine, and pregnancy [3–5]. As a result, the incidence of PDPH vary widely in the literature, depending on the characteristics of the studied populations, and the different applied techniques. However, it was estimated that around one-third of the procedures can be complicated with headache [3, 6].

The exact mechanism of headache in PDPH is still uncertain, and several theories have been proposed. The most common theory is downward stretch of pain sensitive structures when patients assume an upright position, secondary to cerebrospinal fluid (CSF) volume loss [7]. Other theories include hypersensitivity to substance P, compensatory vasodilation of intracranial blood vessels in order to maintain a constant intracranial volume (Monro-Kellie doctrine), and relative CSF hypovolemia resulting from persistent CSF leakage of CSF causing an orthostatic type of headache [8].

In this study, we aimed to evaluate the incidence, risk factors and clinical characteristics of PDPH in the inpatient setting of the main tertiary neurology hospital in Kuwait. To our knowledge, this work represents the first comprehensive description of PDPH from a population of the Arabian Gulf Region.

## Methods

We conducted a prospective observational cohort study that included all patients who were admitted to the neurology department at Ibn Sina hospital, Kuwait, from January 1, 2019 to December 31, 2020, on whom LP was performed for diagnostic and/or therapeutic reasons. Patients who agreed to participate were included consecutively upon admission to hospital, following their voluntary and informed consent. The study was approved by the Scientific Research Committee of Department of Neurology, Ibn Sina hospital, Kuwait.

Demographic data included age, gender, body weight, body height, and BMI. All patients underwent complete history taking, detailed neurological examination, routine laboratory investigations (complete blood count, renal function tests, liver function tests, electrolytes, coagulation profile) and brain imaging (magnetic resonance imaging or computed tomography) prior to LP.

As per department protocol, all the procedures were standardized using a *22*-gauge, atraumatic, non-cutting spinal needle. LPs were performed in either left lateral recumbent or sitting positions. All patients received local injection of lidocaine 1% or 2%, and the procedure was performed in a midline approach, at either L4–L5, or L3–L4 intervertebral space levels. As a standard of care, LP technique included inserting the needle bevel parallel to the dural fibers, passively withdrawing CSF, and replacing the stylet before withdrawing the spinal needle. CSF opening and closing pressures were measured using manometer in lateral recumbent position with the legs relaxed and partially extended, regardless of initial position of the procedure. Routine CSF analysis including levels of protein, glucose, red blood cells (RBCs), and white blood cells (WBCs) was done in all cases.

In the postprocedural period, all patients were instructed to rest in bed for 4 h and they could walk afterwards if their clinical condition permitted. PDPH was assessed on the day of the procedure, upon discharge, and on day 7 and day 14 after LP, either in-person or by telephone, by the study investigators (AR, FM, III). PDPH was diagnosed according to the ICHD-3 diagnostic criteria [2].

Headache-related information included: presence of pre-procedural headache, history of previous PDPH, headache onset, type, duration, and severity. Headache severity was evaluated using Numerical Rating Scale (NRS). Patients self-reported the severity of headache on a scale from (0 to 10); scores from 1 to 3 were interpreted as mild headache, scores from 4 to 7 were considered moderate, and scores from 8 to 10 were considered as severe headache. All patients who developed PDPH were offered medical treatment, and were advised to visit their treating physician in case of severe and/or persistent headaches.

### Statistical analysis

Data were analyzed using IBM SPSS software package version 25.0 (Armonk, NY: IBM Corp). Qualitative data were described using number and percent. The Kolmogorov–Smirnov test was used to verify the normality of distribution. Quantitative data were described using range (minimum and maximum), mean, standard deviation, and median. Chi-square test (Fisher or Monte Carlo), and student t-test were used to compare two groups for normally distributed quantitative variables. Mann–Whitney test was used to compare between two groups for not normally distributed quantitative variables. All of the variables that were statistically significant in univariate logistic regression model were entered into multivariate logistic regression model to explore the factors that are independently associated with development of PDPH. The multivariate model contained variables that were associated with *p* < 0.1 in univariate analysis. Significance of the obtained results was set at *p* ≤ 0.05 level.

## Results

A total of 285 consecutive procedures were performed in our study during the assigned period (225 females (78.9%), mean age of 32.9 ± 11.7 years, and mean BMI of 33.5 ± 37.6 kg/m^2^). Of them, 139 patients (48.8%) had idiopathic intracranial hypertension (IIH), 90 (31.6%) had demyelinating diseases, 26 (9.1%) had peripheral neuropathy (PN), 8 (2.8%) had optic neuritis (ON), 5 (1.8%) had dementia, 4 (1.4%) had cerebral venous thrombosis (CVT), 3 (1.1%) had autoimmune encephalitis, and only 1 patient (0.4%) had CNS infection. Nine patients (3.2%) had spinal muscular atrophy (SMA) and underwent intrathecal treatment with nusinersen (Spinraza ®) 12 mg/5 ml after withdrawing 5 ml of CSF.

PDPH was reported by 84 patients (29.5%). Mean headache onset was 1.7 ± 0.8 days, with 85.7% having headache onset in the first 2 days. Mean headache duration was 2.4 ± 2.1 days. The commonest headache type was dull aching in 49 patients (58.3%%), followed by throbbing in 25 (29.8%), and other types in 10 patients (11.9%). Headache severity was mild in 34 (40.5%), moderate in 30 (35.7%), and severe in 20 patients (23.8%). Mean pain intensity using NRS was 4.1 ± 0.9. Headache complaint prior to LP was reported by 212 patients (74.4%), while history of previous PDPH was reported by 24 patients (8.4%).

All patients with PDPH were offered medical treatment (e.g. bed rest, oral hydration, paracetamol, caffeine), with only 2 patients (0.7%) with persistent headaches needed an epidural blood patch.

As regards to LP, it was performed in left lateral position in 241 patients (84.6%), and in sitting position in 44 patients (15.4%). L4–L5 intervertebral space level was used in 216 patients (75.8%), while L3–L4 intervertebral space level was used in 69 patients (24.2%). Median number of LP attempts was 2, ranging from (1 – 6). LP was performed by a registrar in 189 patients (66.3%), senior registrar in 79 (27.7%), assistant registrar in 13 (4.6%), and consultant in 4 patients (1.4%). Intravenous fluids were given after LP for only 34 patients (11.9%).

As regards to CSF, mean opening pressure was 272 ± 145.6 mm H_2_O, mean closing pressure was 174.9 ± 40.3 mm H_2_O, and mean amount of withdrawn CSF was 8.4 ± 4.3 ml. Mean CSF protein was 296.6 ± 113.2 mg/mL, mean CSF glucose was 4.1 ± 0.8 mmol/L, mean CSF RBCs was 31.5 ± 142.6, and mean CSF WBCs was 4.5 ± 5.7 cells/µL. The demographic and clinical characteristics of our cohort are shown in Table 1.

**Table 1:** Distribution of the studied cases according to different clinical parameters (*n* = 285)

	**No. (%)**
**Age (years)**	
Mean ± SD	32.9 ± 11.7
Median (Min. – Max.)	32 (12 – 75)
**Gender**	
Male	60 (21.1%)
Female	225 (78.9%)
**BMI (kg/m**^**2**^**)**	
Mean ± SD	33.5 ± 37.6
Median (Min. – Max.)	30.4 (21 – 657)
**Diagnosis**	
IIH	139 (48.8%)
Optic neuritis	8 (2.8%)
Peripheral neuropathy	26 (9.1%)
Demyelinating disease	90 (31.6%)
SMA	9 (3.2%)
Autoimmune encephalitis	3 (1.1%)
CNS infection	1 (0.4%)
Dementia	5 (1.8%)
CVT	4 (1.4%)
**PDPH**	
No	201 (70.5%)
**Yes**	**84 (29.5%)**
**Headache onset (days)**	**(*n* = 84)**
1	40 (47.6%)
2	32 (38.1%)
3	10 (11.9%)
4	2 (2.4%)
Mean ± SD	1.7 ± 0.8
Median (Min. – Max.)	2 (1 – 4)
**Headache type**	**(*n* = 84)**
Dull aching	49 (58.3%)
Throbbing	25 (29.8%)
Other	10 (11.9%)
**Headache severity**	**(*n* = 84)**
Mild	34 (40.5%)
Moderate	30 (35.7%)
Severe	20 (23.8%)
**Headache duration (days)**	**(*n* = 84)**
Mean ± SD	2.4 ± 2.1
Median (Min. – Max.)	2 (1 – 14)
**Pain intensity (NRS)**	
Mean ± SD	4.1 ± 0.9
Median (Min. – Max.)	4 (1 – 8)
**Pre-LP headache**	
No	212 (74.4%)
Yes	73 (25.6%)
**History of previous PDPH**	
No	261 (91.6%)
Yes	24 (8.4%)
**LP Position**	
Left lateral	241 (84.6%)
Sitting	44 (15.4%)
**CSF protein**	
Mean ± SD	296.6 ± 113.2
Median (Min. – Max.)	288 (100 – 755)
**CSF glucose**	
Mean ± SD	4.1 ± 0.8
Median (Min. – Max.)	3.7 (2.6 – 6.4)
**CSF WBCs**	
No	257 (90.2%)
**Yes**	**28 (9.8%)**
Mean ± SD	4.5 ± 5.7
Median (Min. – Max.)	2 (1 – 22)
**CSF RBCs**	
Mean ± SD	31.5 ± 142.6
Median (Min. – Max.)	7 (0 – 1236)
**LP location**	
L4–L5 intervertebral space level	216 (75.8%)
L3–L4 intervertebral space level	69 (24.2%)
**CSF**	
**Opening pressure**	
Mean ± SD	272 ± 145.6
Median (Min. – Max.)	220 (95 – 990)
**Closing pressure**	
Mean ± SD	174.9 ± 40.3
Median (Min. – Max.)	170 (90 – 300)
**Amount (ml)**	
Mean ± SD	8.4 ± 4.3
Median (Min. – Max.)	10 (5 – 35)
**Post LP fluids**	
No	251 (88.1%)
Yes	34 (11.9%)
**Number of LP attempts**	
Mean ± SD	1.8 ± 1.1
Median (Min. – Max.)	2 (1 – 6)
**Physician**	
Registrar	189 (66.3%)
Senior registrar	79 (27.7%)
Assistant registrar	13 (4.6%)
Consultant	4 (1.4%)

There was a statistically significant association between development of PDPH and younger age (28.3 ± 11.5 years vs 34.8 ± 11.3, *p* < 0.001), female gender (*p* = 0 0.006), low BMI (26.2 ± 3.9 kg/m2 vs 36.6 ± 44.4, *p* < 0 0.001), pre-LP headache (*p* < 0.001), history of previous PDPH (*p* < 0.001), low levels of RBCs in CSF (14.2 ± 168.3 vs 1.8 ± 2.1, *p* < 0.04), and number of LP attempts (3 ± 1.4 vs 1.5 ± 0.8, *p* < 0.001). As regards to clinical diagnosis; a statically significant association with developing PDPH was observed in patients with ON (*p* = 0.009), and CVT (*p* = 0.007), while patients with PN (*p* = 0.011) and SMA (*p* = 0.042) had less PDPH, which was statistically significant. Other parameters were not found to be statistically significant in our cohort. Correlation findings are summarized in Table 2.

**Table 2:** Relation between PDPH and different parameters (*n* = 285)

	**PDPH**		**Test of sig**	**p**
	**No (*n* = 201)**	**Yes (*n* = 84)**		
**Age (years)**				
Mean ± SD	34.8 ± 11.3	28.3 ± 11.5	U = 5367.50 ^*^	< 0.001 ^*^
Median (Min. – Max.)	33 (12 – 75)	26 (13 – 69)		
**Gender**				
Male	51 (25.4%)	9 (10.7%)	*x*^2^=7.659 ^*^	0.006 ^*^
Female	150 (74.6%)	75 (89.3%)		
**BMI (kg/m**^**2**^**)**				
Mean ± SD	36.6 ± 44.4	26.2 ± 3.9	U = 2506.50 ^*^	< 0.001 ^*^
Median (Min. – Max.)	33.6 (23.6 – 657)	24.7 (21– 38.5)		
**Diagnosis**				
IIH	96 (47.8%)	43 (51.2%)	*x*^2^=0.279 *x*^2^=8.207 ^*^ *x*^2^=6.530 ^*^ *x*^2^=0.018 *x*^2^=3.884 *x*^2^=1.267 *x*^2^=2.401 *x*^2^=0.220 *x*^2^=9.708 ^*^	0.597 ^FE^p = 0.009 ^*^ 0.011 ^*^ 0.895 ^FE^p = 0.042 ^*^ ^FE^p = 0.558 ^FE^p = 0.295 ^FE^p = 1.000 ^FE^p = 0.007 ^*^
Optic neuritis	2 (1%)	6 (7.1%)		
Peripheral neuropathy	24 (11.9%)	2 (2.4%)		
Demyelinating disease	63 (31.3%)	27 (32.1%)		
SMA	9 (4.6%)	0 (0%)		
Autoimmune encephalitis	3 (1.5%)	0 (0%)		
CNS infection	0 (0%)	1 (1.2%)		
Dementia	4 (2%)	1 (1.2%)		
CVT	0 (0%)	4 (4.8%)		
**Pre-LP headache**				
No	188 (93.5%)	24 (28.6%)	*x*^2^=131.210 ^*^	< 0.001 ^*^
Yes	13 (6.5%)	60 (71.4%)		
**History of previous PDPH**				
No	196 (97.5%)	65 (77.4%)	*x*^2^=31.133 ^*^	< 0.001 ^*^
Yes	5 (2.5%)	19 (22.6%)		
**LP Position**				
Left lateral	165 (82.1%)	76 (90.5%)	*x*^2^=3.192	0.074
Sitting	36 (17.9%)	8 (9.5%)		
**CSF protein**				
Mean ± SD	311 ± 125.8	262.4 ± 63.8	U = 6743.50	0.312
Median (Min. – Max.)	299 (100 – 755)	250 (159 – 454)		
**CSF glucose**				
Mean ± SD	4 ± 0.9	4 ± 0.7	t = 0.236	0.814
Median (Min. – Max.)	3.7 (2.6–6.4)	3.9 (3.1 – 5.6)		
**CSF WBCs**				
No	182 (90.5%)	75 (89.3%)	*x*^2^=0.106	0.744
**Yes**	19 (9.5%)	9 (10.7%)		
Mean ± SD	3 ± 2.6	7.6 ± 8.9	U = 77.50	0.699
Median (Min. – Max.)	2 (1 – 10)	2 (1 – 22)		
**CSF RBCs**				
Mean ± SD	14.2 ± 168.3	1.8 ± 2.1	U = 1588.50^*^	< 0.046^*^
Median (Min. – Max.)	6 (0 – 1236)	1 (0 – 10)		
**LP location**				
L4–L5 intervertebral space level	148 (73.6%)	68 (81%)	x^2^=1.730	0.188
L3–L4 intervertebral space level	53 (26.4%)	16 (19%)		
**CSF**				
**Opening pressure**				
Mean ± SD	273.3 ± 153.4	268.9 ± 125.9	U = 8248.50	0.759
Median (Min. – Max.)	200 (95 – 990)	300 (110 – 550)		
**Closing pressure**				
Mean ± SD	175.4 ± 39.8	173.5 ± 41.7	t = 0.364	0.714
Median (Min. – Max.)	170 (90 – 300)	170 (110 – 300)		
**Amount (ml)**				
Mean ± SD	8.1 ± 4.1	9.2 ± 4.6	U = 7669.0	0.190
Median (Min. – Max.)	10 (5 – 35)	10 (5 – 30)		
**Post LP fluids**				
No	175 (87.1%)	76 (90.5%)	*x*^2^=0.659	0.418
Yes	26 (12.9%)	8 (9.5%)		
**Number of LP attempts**				
Mean ± SD	1.5 ± 0.8	3 ± 1.4	U = 3106.0^*^	< 0.001^*^
Median (Min. – Max.)	1 (1 – 6)	3 (1 – 6)		
**Physician**				
Registrar	125 (62.1%)	60 (71.4%)	*x*^2^=3.047	0.096
Senior registrar	52 (25.8%)	17 (20.2%)		
Assistant registrar	8 (3.9%)	5 (5.9%)		
Consultant	2 (0.9%)	2 (2.4%)		

*x*^2^ **Chi square test** *MC* **Monte Carlo, t: Student t-test U: Mann Whitney test**

p *p* value for comparing between **the two studied categories**

^*^ Statistically significant at *p* ≤ 0.05

In multivariate logistic regression, younger age (adjusted OR, 1.030; 95% CI, 0.970 –1.093, *p* = 0.001), female gender (adjusted OR, 1.987; 95% CI, 0.152–4.434, *p* = 0 0.001), low BMI (adjusted OR, 0.639; 95% CI, 0.526–0.776, *p* < 0 0.001), pre-LP headache (adjusted OR, 16.084; 95% CI, 3.110–83.191, *p* = 0.001), history of previous PDPH (adjusted OR, 23.32; 95% CI, 0.736–738.794, *p* = 0.001), and number of LP attempts (adjusted OR, 2.940; 95% CI,1.624–5.320, *p* < 0.001) remained statistically significantly associated with PDPH. However, RBCs in CSF (adjusted OR, 0.511; 95% CI, 0.490–3.762, *p* < 0.213), and the different clinical diagnoses in our cohort were not found to be statistically significant in the multivariate model. The detailed results of univariate and multivariate analysis are shown in Table 3.

**Table 3:** Univariate and multivariate logistic regression analysis for PDPH affecting the different parameters (*n* = 285)

	**Univariate**		**Multivariate**	
	**p**	**OR (95%C.I)**	**p**	**OR (95%C.I)**
**Age (years)**	< 0.001 ^*^	0.945 (0.920 – 0.970)	0.001 ^*^	1.030(0.970–1.093)
**Gender**	0.007 ^*^	2.833 (1.324–6.064)	0.001 ^*^	1.987(0.152–4.434)
**BMI (kg/m**^**2**^**)**	< 0.001 ^*^	0.745 (0.690–0.804)	< 0.001 ^*^	0.639(0.526–0.776)
**Diagnosis**				
Optic neuritis	0.014 ^*^	7.654 (1.512–38.737)	0.798	3.959 (0.0–148,238)
Peripheral neuropathy	0.022 ^*^	0.180 (0.042–0.779)	0.923	1.711 (0.0–91,837)
**Pre-LP headache**	< 0.001 ^*^	36.154(17.338–75.387)	0.001 ^*^	16.084 (3.110–83.191)
**History of previous PDPH**	< 0.001 ^*^	11.458 (4.114–31.913)	0.001 ^*^	23.321(0.736–738.794)
**CSF RBCs**	< 0.04 ^*^	0.596 (0.528–0.973)	< 0.213	0.511(0.490–3.762)
**Number of LP attempts**	< 0.001 ^*^	3.166 (2.311–4.338)	< 0.001 ^*^	2.940(1.624–5.320)

*OR* Odd`s ratio, *C.I* Confidence interval, *LL* Lower limit, *UL* Upper Limit

^#^ All variables with p < 0.05 was included in the multivariate

^*^ Statistically significant at *p* ≤ 0.05

Furthermore, analysis of risk factors showed that the incidence of PDPH in patients with a BMI < 30.0 kg/m (39.3%) was higher than in obese patients with a BMI ≥ 30.0 kg/m (60.7%) (adjusted OR, 0.39;95% CI, 0.16–0.95, *P* = 0.04). However, as regards to CSF pressure, there was no statistically significant difference between patients with normal and high CSF pressure at a cutoff value of > 25 cmH2O (adjusted OR, 24.3; 95% CI, 12.6–16.9, *P* = 0.72).

## Discussion

This work aimed to study the incidence, risk factors and clinical characteristics of PDPH in the main tertiary neurology hospital in Kuwait, which has not been comprehensively studied in a population from the Arabian Gulf Region, to the best of our knowledge. LP was a safe and well-tolerated procedure in our cohort, with no reported serious complications.

### 1- Incidence

In this study, 29.5% of our patients developed PDPH. The reported incidence of PDPH is variable in the literature, ranging from 10 to 40%, depending on several procedural and non-procedural aspects [9, 10]. Historically, higher incidence was reported in older studies, probably secondary to the use of large gauge, cutting spinal needles. PDPH incidence significantly dropped from 66% in a study from 1898 [11], to around 11% [12], after the development of smaller gauge needles and the modifications of spinal needle tips.

Our findings were similar to the values reported in other regional studies, where PDPH occurred in 27.6% [13], and 28.7% [14] of patients, although cutting needles with different gauge sizes were used in these studies. However, in a similar study using 22G or 24G non-cutting needles, the incidence of PDPH was 21.6% during the immediate post procedural period, and 17.5% at 24-h follow-up [15]. On the other hand, in a 2018 meta-analysis of 110 studies [16], the rate of PDPH with a 20–22 G needle was found to be 11.0% when using traumatic needle group compared to 4.2% with atraumatic needles, signifying a 60% reduction in incidence.

This higher incidence in our study could be understood in the light of the clinical characteristic of our patients, rather than procedural-related factors. The majority of our patients had well-documented risk factors such as; female gender, low BMI, and headaches prior to LP. Moreover, in the aforementioned meta-analysis, participants tended to be older (mean age was 38.6 years), with less female participants (61.7%) than our study. Another reason could be the nature of our prospective study, where we evaluate patients after LP and specifically ask if they have headache. Many PDPH studies are retrospective, thus only patients who report headaches are included.

### 2- Headache features

The majority of headaches (85.7%) started within the first 2 days of the procedure, and lasted for average of 2.4 days. This is similar to findings in the literature, where around 66% of PDPH start within the first 48 h of LP, and nearly 90% within the first 72 h [17]. However, headache duration in our cohort seemed shorter than other studies, in which, only a quarter of patients improve within 2 days of onset, and more than half of patients improve by day 4 [18]. This could be related to the mild/moderate severity of the headaches in the majority of our patients, with good response to conservative medical management. None of our patients developed PDPH after 4 days of LP, which is similar to the literature, as PDPH rarely develops between 5 and 14 days after the procedure [18].

The commonest headache type was dull aching in 58.3%, followed by throbbing in 29.8% of patients. In a review of literature [1], the commonest headache types were reported to be dull aching, throbbing, or burning, similar to our findings. However, they reported that pain in PDPH is usually severe, which contradicts our findings, where the majority in our cohort (76.2%) had headaches of mild to moderate severity, with mean NRS of 4.1 ± 0.9.

Moreover, PDPH was self-limited in the majority of patients, with only 2 patients (0.7%) requiring a blood patch. These values compare favorably to published studies of PDPH. In a study by de Almeida and colleagues [19], PDPH was well-tolerated, and only 0.4% of their cohort needed a blood patch.

### 3- Risk factors

The current study supports some earlier findings for PDPH risk factors in the literature, including young age, female gender, low BMI, headache before LP, previous history of PDPH, and number of attempted punctures [4, 20].

#### (a) Age & gender

Young age and female gender are the most important well-documented risk factors for development of PDPH [6, 7, 21]. In our cohort, the mean age of patients with PDPH was 28.3 ± 11.5 years, which was 6.5 years younger than those without headaches. Several studies found that females aged less than 40 years had around 3–5 times higher risk for developing PDPH [17], which was in line with Amorim and colleagues’ findings [22], where incidence was higher between the age of 31 and 50 years. PDPH over the age of 60 is rare, and studies have shown consistent reduction in PDPH incidence with advanced age [20].

Female gender presented a risk for PDPH 8-times greater than that of males, which was found to be higher than reported numbers in the literature. Amorim and colleagues [22] reported 2.25 times greater risk in females, which was similar to a meta-analysis by Wu and colleagues [23], who found that the risk was twice in females, irrespective of other parameters. Flaatten and colleagues [24] reported 3 times risk regardless of age.

The possible underlying etiologies for age and gender differences in PDPH include; differences in pain perception, differences in elasticity of the dura, psychosocial factors, and hormonal effects on the reactivity of cerebral blood vessels [23, 25].

#### (b) BMI

Lower BMI in our study was found to be an independent risk factor for PDPH, similar to other earlier reports in literature. Faure and colleagues [26], reported that in patients with BMI less than 30 kg/m^2^, the percentage of PDPH was 45%, in comparison to patients with BMI greater than 30 kg/m^2^where the incidence was 25%, in a study of 99 patients who had accidental dural puncture. Another study by Peralta and colleagues [27], which examined 13,2013 parturient who received spinal anesthesia, found PDPH incidence to be 56% of in patients with BMI less than 31.5 kg/m^2^, and 39% if the BMI was greater than 31.5 kg/m^2^ (*P* = 0.0004).

Furthermore, morbid obesity was found to decrease the incidence of PDPH in several reports [28]. The proposed mechanisms underlying the association between BMI and PDPH risk are still debatable, however, higher intra-abdominal pressures associated with higher BMI might help in sealing dural tears made during LPs.

#### (c) Headache prior to LP

In our cohort, the majority of patients had headache prior to LP, and more than 70% of them developed PDPH, with a statistical significance. This was similar to the findings by Kuntz and colleagues [28], who found that PDPH incidence in patients with headache 1 week before the procedure was close to 70%, in comparison to 30% incidence in patients without headache. Furthermore, another study [13] found that history of headache was reported in 67% of patients with PDPH, compared to 38% in the patients without PDPH, in the general population. One possible explanation could be related to nociceptive central sensitization in patients with headache prior to LP, which leads to higher risk of developing PDPH after LP [29].

#### (d) History of previous PDPH

A previous history of PDPH was estimated to have 2–3 times higher risk for developing a new attack of PDPH in several reports, compared to patients who never developed PDPH before [20, 30]. Amorim and colleagues [7], found that 19% of patients with prior history of PDPH developed second PDPH, which was similar to our findings, where 22.6% of patients with prior PDPH developed new attack of PDPH. This increased incidence of PDPH with history of previous PDPH is likely related to the underlying clinical characteristics of this group of patients, which predisposes them to develop another PDPH [22].

#### (e) Number of LP attempts

Higher number of LP attempts increased the risk of PDPH in our study. It has been shown that PDPH is more common if two verified punctures into the subarachnoid space are made [31]. A prospective study on 8,034 patients who underwent LP during spinal anesthesia, confirmed that repeated punctures increased the incidence of PDPH [32].

### 4- Possible effect of the underlying clinical diagnosis

In this study, PDPH was statistically significantly higher in patients with ON and CVT, and lower in patients with PN and SMA, although this association was not confirmed in the multivariate regression analysis. This could be attributed to other clinical characteristics in those patients, such as the presence of headache prior to LP, or the predominance of female gender in patients with CVT and ON, in our cohort.

Several studies in the literature reported variable effects of different clinical diagnoses on the development of PDPH. Similar to our findings, dementia [33, 34] and SMA [35] showed low incidence of PDPH in the literature. Interestingly, none of our SMA patients had PDPH, which could be attributed not only to the small number of patients, but might also be related to the removal of small amount of CSF and immediate replacement with the drug during the procedure. Other studies reported higher incidence of PDPH in patients with IIH [36], and MS [37], with an incidence of 23.0% and 57%, respectively.

### 5- Other factors not associated with PDPH

In our study, CSF opening pressure, closing pressure, CSF volumes or constituents, were not associated with the development of PDPH, similar to several previous reports [19, 38, 39]. Interestingly, higher numbers of RBCs in CSF were found to be inversely correlated with developing PDPH, however, this finding was not confirmed in multivariate analysis. One possible explanation is that the coagulating effect of the blood can act as a patch for the dural tear created during LP, which was observed in few reports [40]. However, other studies failed to find significant correlation of PDPH and RBC counts [19].

Moreover, the lack of association of LP location, position, duration of rest after LP, and post-LP fluids and PDPH in our cohort, was in line with findings in the literature [13, 19, 41]. Furthermore, the experience of the neurologist was not correlated with developing PDPH in our study. In literature, there is no strong evidence that correlates physician’s experience and PDPH. Our findings matched other studies, where there was no effect of operator experience on developing PDPH after 300 diagnostic LPs in one study [21], and after 501 LPs in another [28]. However, this finding contradicts other earlier reports. In one study [42], the incidence of PDPH was 2-times higher with less-experienced anesthesiologists compared to experienced ones (2.5% vs 1.2%). This was also similar to another study [43], where the most junior clinicians had double the incidence of PDPH when compared to consultants, although this finding was not statistically significant (0.2% vs 0.13%, *p* > 0.05). One can argue that this lack of correlation is attributed to the lesser degrees of variability in the technical performance of the LPs, as all neurologists in the department have more than 3 years of experience, under close supervision and rigorous training.

### 6- Treatment

In literature, more than 85% of PDPH resolves with conservative measures, or without any specific treatment [25]. Despite the lack of evidence on pharmacological management of PDPH [44], the majority of our patients were managed by conservative measures that included bed rest in supine position, oral or intravenous hydration, caffeine supplementation, and simple analgesics, with good response in the majority of them. Only 2 patients had persistent headaches that required epidural blood patch, which is similar to other published values [45, 46].

### Strength and limitations

This study has some limitations. First, the results were obtained from a single-center, and the sample size was not calculated according to a predefined outcome. However, we included all consecutive eligible patients admitted to the largest neurology tertiary hospital in Kuwait, during the study period, which also minimized the possibility of selection bias. Few studies in the literature present a uniform data set regarding LPs and PDPH, as most studies use different techniques and protocols. Moreover, our findings came in line with published data on PDPH, which further validates our sample, and confirms that our cohort is representative to the population of patients undergoing LP in neurology departments.

Secondly, given our department standardized protocol for LPs, we could not evaluate the impact of certain procedural factors such as; needle type, needle size, bevel direction, and non-replacement of the stylet, on the development of PDPH. Moreover, LPs in the study were performed by physicians of varying experience, which could have affected the results. However, all of them have received vigorous training for the procedure, and we aimed to assess this variation on the primary outcome, as this represents the real-life clinical situation in most hospitals.

## Conclusions

Our findings from clinical practice in the main tertiary neurology hospital in Kuwait were in line with literature data regarding PDPH. To our knowledge, this study represents the first comprehensive description of PDPH from the Arabian Gulf Region.

In the majority of patients, headache started within the first 2 days of the procedure, lasted for average of 2.4 days, was dull aching or throbbing in nature, and of mild to moderate severity. Independent risk factors included younger age, female gender, lower BMI, pre-procedural headache, previous history of PDPH, and number of LP attempts. Identification of risk factors is important in predicting PDPH, and preventing this painful adverse event should be the main goal of neurologists dealing with this population. These results should be validated in further multicenter studies with larger cohorts to confirm our findings.

## Data Availability

The data generated for this study are available on request to the corresponding author.

## Abbreviations

PDPH: Post-dural puncture headache
ICHD-3: International Classification of Headache Disorders, 3^rd^ edition
LP: Lumbar puncture
BMI: Body mass index
CSF: Cerebrospinal fluid
RBCs: Red blood cells
WBCs: White blood cells
NRS: Numerical Rating Scale
IIH: Idiopathic intracranial hypertension
PN: Peripheral neuropathy
ON: Optic neuritis
CVT: Cerebral venous thrombosis
SMA: Spinal muscular atrophy
